# Stretching of the Lingual Nerve for Neuralgia

**Published:** 1885-05

**Authors:** 


### Stretching of the Lingual Nerve for Neuralgia.

Mr. Clement Lucas operated for the relief of extreme neuralgia of the tongue at Guy's Hospital, November 11th, by stretching the lingual or gustatory nerve. He pointed out what he believed to be an original observation: that if the tongue be seized by the tip and drawn forcibly out of the mouth and on one side, the lingual nerve of the opposite side is made to stand out as a firm band beneath the mucous membrane on the side of the tongue, where it can be readly felt and secured. The operation was performed as follows: A suture was placed through the tongue to the right of the septum, by means of which the organ was drawn forcibly out and to the left side. A sharp-pointed hook was then passed under the nerve to fix it. The mucous membrane was then divided over the nerve for about half an inch, so that it could be readily seen, and an aneurism-needle having been passed immediately under the nerve, the sharp hook was withdrawn. The nerve was in this way easily reached and stretched.—*British Med. Jour.*

